# The Influence of Popular Media on Public Interest in Red-Light Therapy: Longitudinal Trend Analysis

**DOI:** 10.2196/69796

**Published:** 2025-06-12

**Authors:** Catherine Z Shen, Aaron T Zhao

**Affiliations:** 1 Perelman School of Medicine, University of Pennsylvania, 3400 Civic Center Blvd, Philadelphia, PA, 19104, United States, 1 240-396-6874

**Keywords:** social media, dermatology, trends, red-light therapy

## Abstract

TikTok’s influence has significantly increased public interest in red-light therapy, surpassing that for traditional skin care treatments; this highlights the powerful role of social media in shaping health care trends and underscores the need for health care providers to stay informed about viral social media trends on treatment.

## Introduction

The intersection of social media and health care information dissemination has created new challenges and opportunities for health care professionals. Social media platforms, particularly TikTok, increasingly shape public interest in medical treatments. In early 2024, red-light therapy (RLT) emerged as a viral skin care trend on TikTok; celebrities featured LED masks from brands such as Omnilux in their content. By February 2024, the hashtag “Red LED light therapy” had >70 million views on TikTok, driving interest in home-use devices ranging in price from US $100 to $3500 [1]. This attention came despite limited scientific understanding of the long-term effects and safety, especially for home use [2,3].

RLT, also known as photobiomodulation or low-level laser therapy, is purported to have beneficial effects on skin health [4]. While some clinical applications of this therapy are well documented, recent interest primarily focuses on consumer-grade devices and home treatments, raising concerns among health care providers about safety and efficacy [5]. Here, we examine the impact of TikTok exposure on public interest in RLT and compare trends with conventional skin care treatments.

## Methods

### Overview

We analyzed Google Trends data from November 2019 to November 2024 for terms related to RLT (“light therapy,” “red therapy,” “red light masks,” “red therapy benefits,” “photobiomodulation,” “low level laser therapy”) and control terms representing traditional skin care treatments (“chemical peel,” “skin care,” “exfoliation”) selected systematically based on preliminary TikTok hashtag analysis and existing literature on light therapy terminology. RLT-related terms were chosen based on their relevance to clinical applications and consumer terminology used on social media. Control terms were related to traditional skin care treatments with comparable market presence to provide appropriate comparison baselines.

Statistical analyses included trend analysis using linear regression and Mann-Kendall tests, with structural breaks identified using Chow tests. All analyses used Python 3.13.0, with significance set at *P*<.05. Artificial intelligence tools helped generate visual representations of search trends over time.

### Ethical Considerations

The University of Pennsylvania waived institutional review board approval for this study as it exclusively used deidentified, publicly available data.

## Results

**Figure 1.**
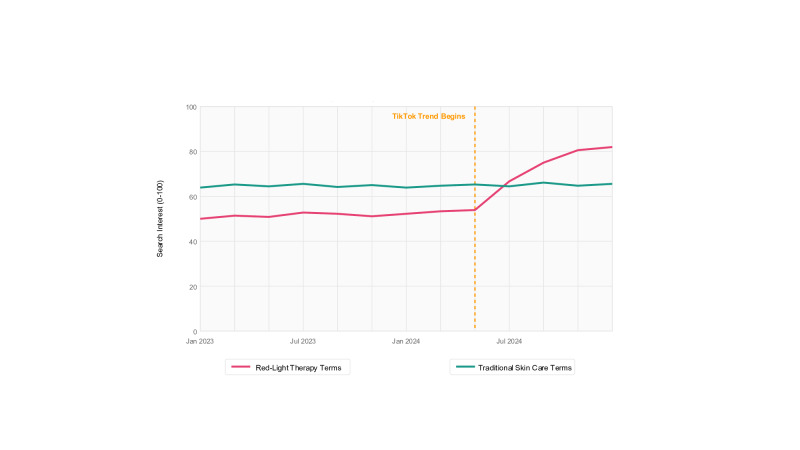
Google Trends search interest for red-light therapy versus traditional skin care terms (2023‐2024), illustrating the substantial increase in search interest for red-light therapy terms compared to traditional skin care terms following viral TikTok exposure in February 2024. Red-light therapy terms included “red light masks,” “light therapy,” and “red therapy.” Control terms included “skin care,” “exfoliation,” and “chemical peel.”

All RLT-related terms, except “low level laser therapy” (*P*=.30), showed significant increases in search volume after February 2024, with the average search volume increasing from 27.8 to 60.5 searches per term (118% increase) compared to baseline (*P*<.001). Figure 1 shows the dramatic increase in searches for RLT terms compared to control terms. Table 1 presents a statistical analysis of key terms.

**Table 1.** Analysis of red-light therapy search terms from November 2019 to November 2024.

Search term	Linear regression slope	*R* ^2^	Mann-Kendall τ	Structural break (*P* value)
“Red light masks”	0.08	0.45	0.38	<.001
“Light therapy”	0.09	0.62	0.58	<.001
“Red therapy”	0.15	0.72	0.68	<.001
“Skin care”	−0.01	0.03	−0.11	—^a^
“Exfoliation”	−0.02	0.04	−0.14	—
“Chemical peel”	−0.008	0.009	−0.07	—

a Not applicable.

Linear regression revealed significant positive trends for RLT terms (slopes: 0.08‐0.15; all *P*<.001), while control terms showed either no significant trends or slight declines. Mann-Kendall tests confirmed strong upward trends for RLT-related terms (τ=0.38‐0.68; all *P*<.001). Structural breaks occurred in early 2024 (all *P*<.001), coinciding with TikTok exposure.

## Discussion

Public interest in RLT significantly increased in early 2024 following its viral popularity on TikTok; by February 2024, “Red LED light therapy” amassed >70 million views [1]. This surge in interest presents both opportunities and challenges for dermatology practitioners and the broader medical community. The rapid adoption of consumer-grade devices, often supported by inconsistent treatment protocols, raises concerns about patient safety and the need for professional oversight.

Average search volume for chemical peels, a traditional treatment, slightly decreased (from 25.8 to 25.5, a −1.2% change; *P*=.09) despite projected market growth, suggesting a shift in consumer interest toward emerging technologies rather than a true decline in established treatments.

Although current research supports RLT for targeted conditions such as wound healing, inflammatory acne, and photoaging, social media claims often amplify its benefits beyond available evidence [6-10]. This underscores the need for additional randomized controlled trials, particularly studies examining the efficacy and safety of consumer devices.

Health care providers must address patients’ growing interest in RLT while maintaining high standards of clinical care. This requires developing structured approaches to patient education that address common misconceptions and provide evidence-based guidelines.

The phenomenon has broader implications for public health trends. Improved regulation of consumer devices, standardized safety guidelines, and enhanced adverse event–reporting systems are urgently needed. Public health communication must evolve to address viral trends, particularly those involving medical devices or treatments. Rapid dissemination of medical information on social media presents opportunities and challenges, highlighting the need for responsive professional education and communication strategies.

Study limitations include the broader scope of RLT keywords (which encompass both dermatologic and other applications) compared to the primarily dermatologic control terms, though the analysis focused specifically on consumer interest rather than clinical equivalence between treatments. Google Trends data also do not reflect actual treatment use or capture detailed demographic data. Future research should incorporate sales data, adverse event reports, and clinical outcomes to comprehensively explore this phenomenon.

These findings have significant implications for dermatology and patient care. As social media continues to shape health care trends, medical professionals must adapt education approaches while maintaining evidence-based practices. This may require new frameworks for addressing viral health care trends and improved methods for communicating scientific evidence. The rapid rise in RLT interest is a case study in how social media can quickly transform patient interest and treatment expectations, requiring the medical community to respond with agility and scientific rigor.

## Abbreviations

RLT: red-light therapy
